# Impaired light detection of the circadian clock in a zebrafish melanoma model

**DOI:** 10.1080/15384101.2015.1014146

**Published:** 2015-04-02

**Authors:** Noémie Hamilton, Natalia Diaz-de-Cerio, David Whitmore

**Affiliations:** Center for Cell and Molecular Dynamics; Department of Cell and Development Biology; University College London; London, UK

**Keywords:** cancer, cell cycle, circadian clock, light input, melanoma, zebrafish

## Abstract

The circadian clock controls the timing of the cell cycle in healthy tissues and clock disruption is known to increase tumourigenesis. Melanoma is one of the most rapidly increasing forms of cancer and the precise molecular circadian changes that occur in a melanoma tumor are unknown. Using a melanoma zebrafish model, we have explored the molecular changes that occur to the circadian clock within tumors. We have found disruptions in melanoma clock gene expression due to a major impairment to the light input pathway, with a parallel loss of light-dependent activation of DNA repair genes. Furthermore, the timing of mitosis in tumors is perturbed, as well as the regulation of certain key cell cycle regulators, such that cells divide arhythmically. The inability to co-ordinate DNA damage repair and cell division is likely to promote further tumourigenesis and accelerate melanoma development.

## Introduction

Most organisms possess a highly conserved endogenous circadian clock, providing a clear survival advantage to animals that live under an environmental light and dark cycle.^1^ The molecular clock mechanism operates through a transcription-translation negative feedback loop of circadian genes and proteins. The alternating activation and suppression of core clock genes, such as *Clock*, *Bmal*, *Per* and *Cry*, produce a 24 h oscillation, which can then regulate the timing of a wide range of downstream, output processes.^2,3^ One of the most significant of these outputs is the daily control of cell proliferation and DNA repair, which has been shown in numerous tissues across many animal model systems.^4-7^ We have previously reported that this cellular clock controls cell cycle events in normal proliferative tissues from early development until adulthood in zebrafish. This cellular clock also controls the timing of the cell cycle in cell culture, with S-phase occurring in the late day/early evening and mitosis in late night/early morning.^6,7^ The clock regulation of key cell cycle regulators, such as the inhibitors *p21* and *p20*, creates a window or gate that permits cells to enter S-phase from G1 when expression levels are low.^8^

Circadian clock disruption is associated with numerous health problems and has been linked to an increased incidence of cancer.^9^ Epidemiologic studies, for example, have revealed an increased risk of breast and colorectal cancers in night shift workers.^10-12^ The hypothesis of a disrupted clock involved in cancer development has also been supported by studies in rodents. Mice, whose clock had been surgically disrupted by removal of the suprachiasmatic nucleus, and then inoculated with tumor cells showed an increase in tumourigenesis.^13^ Environmental clock disruptions by constant light or jet lag exposure in rats and mice have also revealed an increase in spontaneous tumor appearance and tumor growth.^13-15^ Several clock genes have been proposed to act as tumor suppressors and the PER family of proteins in particular appears to play a role in DNA damage repair and tumor suppression. Over expression of *Per1* in colon cancer cell lines was associated with a higher level of apoptosis after irradiation, whereas inhibition of *Per1* expression led to a decrease in apoptosis.^16^ Similar effects were observed with *Per2* in a leukemia cell line.^17^ Transgenic mice lacking both *Per1* and *Per2* showed higher rates of tumourigenesis after irradiation.^18^ Moreover cancer patients commonly show disruptions in their circadian clock, which is nowadays being used as a prognostic tool for breast cancer patients.^19^ Chronochemotherapy has emerged as a treatment strategy, with the discovery of a circadian profile for drug target genes, including those involved in the cell cycle. This mode of treatment takes advantage of the asynchrony between healthy and cancerous tissues and has proven to be successful in delivering treatment at an optimal time of day to increase survival of colorectal cancer and childhood acute leukemia patients compared to normal, non-timed protocols.^20-22^

Melanoma is a very severe and significant form of skin cancer, with approximately 20% of diagnosed individuals succumbing to the disease. It is also a form of cancer that is showing the most dramatic increase in incidence within the population. This number is set to rise even further in Western countries, as travel to sunny locations and exposure to DNA-damaging UV light has escalated in recent years.^23,24^ Human skin is very sensitive to light exposure, especially in the UV range, which can lead to DNA damage and the initiation of melanoma.^25,26^ Skin cells have been shown to contain a robust biological clock in mammals although the function of this skin clock is generally unknown.^27-30^ There is evidence for a role of the circadian clock in maintaining stem cell heterogeneity in the epidermis and the timing of DNA replication appears to be under clock-control in keratinocytes.^31,32^ Analysis of clock gene expression in human skin and melanoma tumor biopsies showed down regulation in tumor samples.^33^ However, the circadian profile of clock genes and clock-controlled genes (CCGs) remains unexplored in melanoma. How clock-cell cycle interactions function in a melanoma tumor environment is an important issue and one of considerable clinical significance.

Zebrafish have already proven to be an excellent vertebrate system in which to study melanoma, due in part to the high gene homology to mammals in cancer related pathways.^34,35^ Using a zebrafish melanoma model, we have analyzed the circadian profile of clock gene expression over several days *in vivo* and *in vitro* and observed a down regulation of clock gene expression in melanoma tumors compared to healthy skin. We have shown that impaired light detection in melanoma tumors may underpin the disruptions observed in central circadian clock components. It is also clear that the circadian timing of mitosis itself is disrupted in these tumors, along with corresponding changes in gene expression. Loss of light detection also compromises induction of the DNA-damage repair pathways, a fact that may promote further cellular mutations, and promote additional, accelerated tumor growth.

## Results and Discussion

### Clock gene expression is altered in zebrafish melanoma tumors

To explore the expression profile of clock genes in melanoma we used transgenic zebrafish *Tg(mitfa:V12Ras)* expressing the constitutively active V12Ras under the control of the melanocyte specific promoter *mitfa*.^35,36^ These animals were crossed to *nacre* (*mitfa*^−/−^) animals to generate a *Tg(mitfa:V12Ras);mitfa^−/^*^−^ strain lacking melanocytes. Injection of a miniCoopR-GFP vector [33] into zygotes from a *Tg(mitfa:V12Ras);mitfa^−/^*^−^ × *mitfa^−/^*^−^ cross was then used to rescue the melanocyte lineage in offspring and induce more penetrant and rapid melanoma development than observed in *Tg(mitfa:V12Ras)* alone. In parallel, the miniCoopR-GFP vector drives expression of GFP in rescued melanocytes.^36^ All *Tg(mitfa:V12Ras);mitfa^−/^*^−^*;*miniCoopR-GFP^+^ (henceforward *V12Ras*^+^; *GFP*^+^) animals developed dysplastic melanocytic pigmentation pattern from day 4, and consequently GFP-labeled melanoma tumors at around 4 weeks of age while their *mitfa^−/^*^−^*;* miniCoopR-GFP^+^ (henceforward *GFP*^+^) siblings showed normal pigmentation and no tumor (**Fig. S1**).

**Figure 1.**
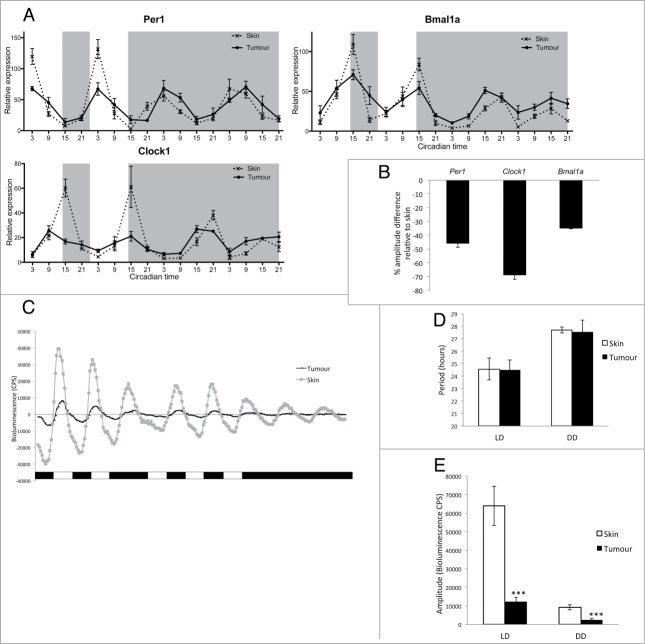
Clock gene expression is downregulated in zebrafish melanoma tumors. (**A**) qPCR analysis of core clock genes *per1*, *clock1*, and *bmal1a* in tumors and skin from animals maintained on a LD cycle, then transferred to DD. Relative expression to the reference gene represents the mean ± SEM of a minimum of 5 samples per time point. White and gray backgrounds represent light and dark phases respectively. (**B**) Quantification of the reduction in amplitude in tumors using skin as a reference. Data represent the mean ± SEM of 5 samples. (**C**) Bioluminescent traces of *per3*-luciferase tumors and skin in LD, then transferred to DD. Results are presented as detrended data from representative samples and white and black bars under the traces represent light and dark phases respectively. (**D**) Period lengths in LD and DD, calculated from the bioluminescent data, are presented in hours. (**E**) Amplitude differences between skin and tumors in LD and DD from the bioluminescent data are presented in counts per second (CPS). Data represent the mean ± SEM of 12 samples using a Student's t-test (unpaired, 2-tailed; ***P < 0.001).

Expression of key clock genes was explored *in-vivo* in melanoma tumors from *V12Ras*^+^; *GFP*^+^ zebrafish and compared to expression in healthy skin harvested from control *GFP*^+^ animals. qPCR analysis showed a significant down-regulation of clock gene expression in tumors compared to healthy skin samples (**Fig. 1A**). Samples were collected every 6 hours over 4 days, 2 days under light-dark (LD) conditions, and 2 subsequent days in constant dark (DD), free-running conditions. In LD, we observed shallow rhythms, with significantly reduced amplitude, for the core clock genes *per1*, *clock* and *bmal1a* in tumor compared to robust rhythms in healthy skin (**Fig. 1A**, **Tables S1, S2**). The significant reduction in amplitude under LD conditions is quantified in **Figure 1B**.

A reduction in circadian clock amplitude has been reported in other types of cancer such as breast, prostate, non-small cell lung cancer, and head and neck squamous cell carcinoma.^37-40^ In particular, a study on human melanoma tumor biopsies has reported a significant down regulation of clock genes compared to adjacent healthy tissues.^33^ However, human skin contains different cell types displaying a range of amplitudes in clock gene rhythms, with keratinocyte and dermal fibroblast cultures showing a more robust clock compared to melanocyte cultures.^28^ To ensure that the difference in amplitude we observed between normal skin and melanoma tumors in our study is not due to a higher number of melanocytes in tumor samples, we analyzed clock gene expression in zebrafish dysplastic naevi (**Fig. S2**). Dysplastic naevi appear during the radial growth phase of melanoma development and lack alterations in certain pathways, such as phosphoinositide 3-kinase (PI3K)-AKT signaling, required for the vertical growth phase and formation of a melanoma tumor.^35^ Naevi, therefore, provide a melanocyte-rich environment, which has not yet progressed to a malignant melanoma (**Fig. S1**). Analysis of *per1*, *clock1* and *bmal1a* expression across one LD cycle showed no down regulation in naevus samples compared to healthy skin (**Fig. S2**). This result indicates that the decreased amplitude in clock gene expression seen in melanoma tumors is most likely due to malignant transformation itself and not to the abundance of melanocytes.

Interestingly, under DD conditions, we found very little disruption of clock gene expression in tumors when measured by qPCR (**Fig. 1A**). However, cosinor analysis of the data revealed a significant increase of the mesor in tumors versus skin but most importantly the amplitude relative to the mesor showed a significant decrease for *per1*, *bmal1a* and *clock* (**Tables S1, S2**), suggesting a defective molecular clock in the tumor population. However, the tumor situation *in vivo* is quite complex in that healthy tissues are surrounding a heterogeneous tumor. To determine the impact of malignancy on clock function in the tumor more precisely it is essential to follow circadian oscillations within single tumors dynamically and *in vitro*. We consequently examined clock rhythms in tissue culture of single tumors and healthy skin controls expressing *period3-luciferase* in bioluminescent assays across 4 days in LD then transferred to DD (**Fig. 1C**). Under these conditions, the period length of the circadian oscillator showed no statistical difference between tumor and skin, either in LD or DD (**Fig. 1D**). However in LD, the amplitude was dramatically reduced in tumors compared to skin (**Fig. 1E**), even more so than in our *in vivo* qPCR analysis (**Fig. 1A, B**). Moreover, the amplitude of tumor circadian rhythms dampens rapidly when transferred to DD compared to data shown *in vivo* by qPCR in **Figure 1A** (**Fig. 1E**). The circadian pacemaker in tumors *in vitro*, therefore, appears more disrupted than *in vivo*, possibly due to the lack of support from surrounding healthy tissue. This suggests that healthy surrounding tissues are likely to play a role in maintaining tumor rhythmicity *in vivo*, though the mechanism of oscillator coupling is far from clear. Nevertheless, we have shown that the circadian clock does continue to function within the melanoma, if with reduced amplitude. This reduction in robustness could be due to a variety of alterations in tumor clock function, including perturbation of the light input pathway, reduced cellular coupling between oscillators or disruption in the regulation of the core clock mechanism within each cell.

### Impaired light detection in the tumor reduces induction of genes involved in clock entrainment and the DNA-damage repair system

**Figure 2.**
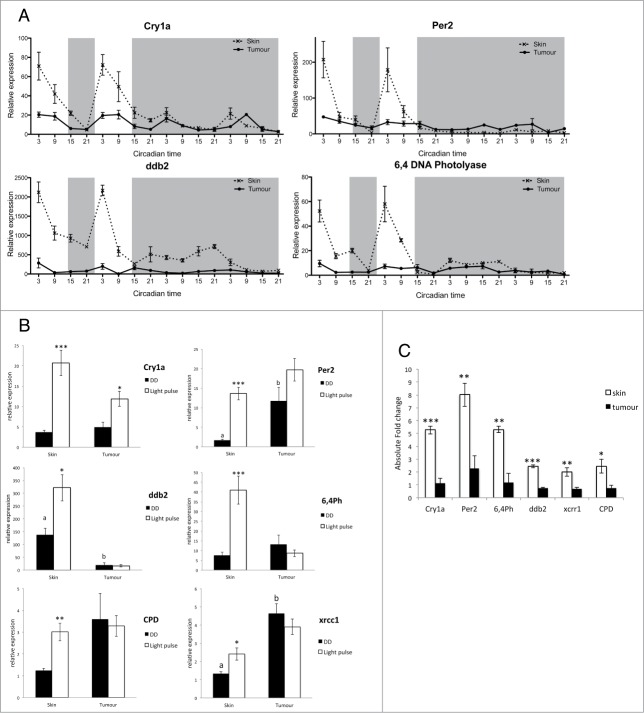
Impaired light detection and DNA-damage repair system. (**A**) qPCR analysis of light inducible clock genes *per2* and *cry1a* and light dependent DNA-damage repair genes *6,4ph* (*6,4 photolyase*) and *ddb2* (*DNA damage binding protein 2*). Relative expression to the reference gene represents the mean ± SEM of minimum 5 samples per time point. (**B**) qPCR analysis *cry1a*, *per2*, *ddb2* and *6,4ph* expression in skin and tumor after a 3 hour light pulse given at CT16 compared to samples kept in the dark (DD). Data represent the mean ± SEM of a minimum of 5 samples per time point. (**C**) Absolute fold induction of each gene in response to light in skin and tumor. Fold induction was compared between skin and tumor using a Student's t-test (unpaired, 2 tailed; **P < 0.01; ***P < 0.001). Data represent the mean ± SEM of 8 samples.

Light is the main entraining signal to the circadian clock, and the majority of zebrafish cells and tissues are themselves directly light responsive.^41^ Two light-inducible clock genes, *cry1a* and *per2,* have been shown to play a critical role on the light input pathway to the clock in zebrafish.^42,43^ We examined by qPCR the expression of these 2 genes *in vivo* in LD and then transferred into DD. *Cry1a* expression was dramatically down regulated in tumor samples in LD, but showed similar levels in DD compared to skin samples (**Fig. 2A**). *Cry1a* expression is under clock control as well as being light inducible, a fact that explains the similar oscillation that continues in constant darkness. The light-inducible clock gene most disrupted in the tumor was *per2* with more than an 80% reduction in amplitude in tumor samples compared to skin (**Fig. 2A**, **Fig. S3**). The dramatic reduction in expression of the 2 genes known to be involved in zebrafish clock entrainment is likely to contribute to the reduced amplitude rhythms in LD shown in **Figure 1**. In many respects, it is an unexpected result that the light input pathway to the clock is so strongly affected in this zebrafish model of melanoma, even when one takes into account the fact that the skin is very strongly light responsive. In mammals there is no evidence at this time that peripheral tissues are themselves directly light sensitive. A detailed analysis of clock function in a mammalian melanoma context is certainly important. However, many of the genes involved in the clock system are highly conserved between zebrafish and mammals, even if there are changes in their specific roles. This is certainly true in the case of both *period 2* and *cryptochrome* genes. There is also considerable evidence that *per2* can act as a tumor suppressor in mammalian systems.^44,45^ Disruption of *per2* expression, either when it is acting as a component of the circadian clock mechanism as in mammals, or the light input pathway as in zebrafish, could potentially play a significant role in enhanced tumourigenesis.

Melanoma tumors often appear black due to the melanin pigment present in melanocytes. To ensure that this extra pigmentation of the tissue does not affect the ability of the tumor to perceive light, we analyzed the expression of *per2* and *cry1a* in naevi, which are also highly pigmented. *Per2* and *cry1a* showed no down regulation of expression in naevi but interestingly a slightly greater amplitude compared to skin samples (**Fig. S4**). We can therefore conclude that there is a defect in the pathway leading to the induction of light responsive genes in melanoma tumors, not resulting from the increased pigmentation, and which may lead to a reduction in the robustness of the clock rhythm measured in these tumors relative to neighboring, healthy tissue.

Transcriptional regulation by light can also directly affect other cellular processes, such as DNA damage repair pathways.^46,47^ Expression of the DNA-damage repair genes *ddb2* and *6,4 photolyase (6,4ph)* displays a robust oscillation in LD peaking 3 h after lights on (Zeitgeber Time—ZT3) in skin samples, which is then lost when tissues are transferred to DD (**Fig. 2A**). However, the expression profile of these genes in tumor samples lacks such oscillations in LD, showing arrhythmic expression and a reduction of more than 80% in expression levels compared to skin samples (**Fig. 2A**, **Fig. S3**). A cosinor analysis of these data confirms that there are no significant rhythms in tumor samples (**Tables S3, S4**), and shows that the loss of light sensitivity impacts not only clock gene expression, but also DNA repair gene induction even more dramatically. Analysis of *ddb2* and *6,4ph* gene expression across a LD cycle in naevi compared to tumor and skin samples showed a gradient of down regulation (**Fig. S5**). This is in agreement with studies on dysplastic melanocytes displaying an impaired DNA damage repair system, creating an environment prone to increased genome instability and allowing for the naevi to progress to melanoma.^48^ This partial loss of light induction in DNA damage repair genes in naevi, at an early stage of melanoma development, establishes a situation that promotes and possibly even accelerates subsequent tumor growth.

To assess the light input pathway in melanoma tumors, we examined the induction of *cry1a*, *per2*, and 4 DNA damage repair genes following a 3 h light pulse during the subjective night (**Fig. 2B**). The expression of all genes was greatly enhanced following the light pulse in control skin samples, as previously reported in zebrafish cell lines and larvae.^42,47^ The acute light response was significantly reduced for *cry1a* expression in tumors, and no significant response occurred at all for *per2* and DNA-damage repair genes (**Fig. 2B**). All light-inducible genes examined showed a significant absolute fold reduction in expression, which demonstrates the profound impairment in the light detection pathway in melanoma tumors (**Fig. 2C**). A functional DNA repair system is essential to protect against mutations caused by UVA and UVB light, especially in skin cells. *ddb2* and *6,4ph* expression in healthy skin was shown to peak at the start of the day therefore providing optimal protection against UV-induced mutations. Melanoma tumors, however, lack light inducible expression of these key DNA damage repair genes, which suggest that their DNA-repair mechanisms will be compromised, and consequently, they are prone to attaining even greater levels of DNA damage and genomic instability.

### Melanoma tumors display disruption in the timing of clock output events

**Figure 3.**
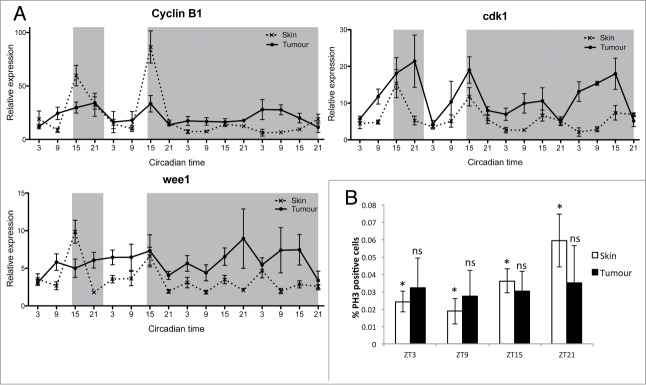
Disruption in mitotic events in melanoma tumors. (**A**) qPCR analysis of mitotic genes *cyclin B1*, cdk1 and *wee1* in healthy skin and tumors. Relative expression to the reference gene represents the mean ± SEM of minimum 5 samples per time point. (**B**) Quantification of mitotic events in healthy skin and tumors using an antibody to phospho-Histone H3 (pH3). Data represent the percentage of pH3-positive cells relative to the total number of cells in one section. Data represent the mean ± SEM of a minimum of 3 different fish. The percentage of pH3 positive cells were compared at each time point for skin and tumor using a one-way ANOVA test followed by a Newman-Keuls multiple comparison post test (*P < 0.05, ns = non significant).

Controlled timing of the cell cycle is one of the major circadian clock outputs. We have previously reported that key cell cycle regulators are clock controlled in zebrafish cell lines and embryos.^6,8,49^ To explore the circadian expression profiles of cell cycle genes in healthy skin and melanoma tumors, we collected tissue samples at 6-hour intervals over LD and DD cycles for 4 days. qPCR analysis of mitotic-related genes (*cyclin B1*, *cdk1*, *wee1*) in skin showed rhythmic expression in LD, with peak expression during the early night (**Fig. 3A**). In comparison, expression in tumor showed clear disruption, including loss of rhythmicity in *wee1* in LD, and amplitude variations in *cyclinB1* and *cdk1*. All mitotic genes showed a loss of rhythmicity in DD (**Fig. 3A**, **Tables S5, S6**), suggesting that the circadian clock might have reduced control over the timing of mitosis. To determine if this disruption in mitosis-related gene expression affected actual mitotic timing, we used a phospho-histone H3 (pH3) antibody to label cells in mitosis in zebrafish skin and tumor sections at 4 different time points across a LD cycle. Skin samples showed a rhythm in mitosis with the highest percentage of pH3 positive cells found during the late night (ZT21) (**Fig. 3B**). Tumor samples did not show any rhythm in mitotic events with a high degree of variability in the percentage of pH3 positive cells at each time point. Interestingly, the average index of mitotic events remained similar throughout the skin and tumor samples, suggesting that the overall number of cells undergoing mitosis is very similar (**Fig. S6**). Most healthy skin cells undergo mitosis at night, possibly to avoid any negative consequences caused by UV light exposure during the day, whereas tumor cells divide randomly at any time of day.

**Figure 4.**
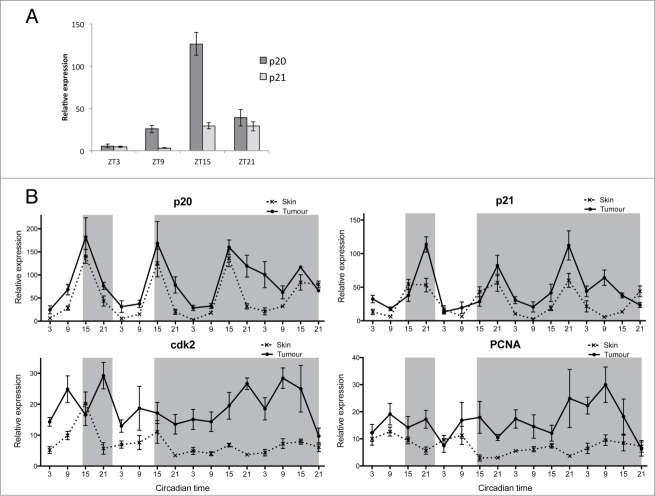
Disruption in S-phase gene expression in melanoma tumor. (**A**) qPCR analysis of S-phase regulating genes *p21*, *cdk2*, and *PCNA* in skin and tumor. Relative expression to the reference gene represents the mean ± SEM of a minimum of 5 samples per time point. (**B**) Cosinor analysis from qPCR values presented in (**A**). Data represent the mesor and amplitude in relative expression, the acrophase in circadian time and the significance of rhythmicity (*P < 0.05; ***P < 0.001) for skin and tumor in LD and DD regimes.

We have hypothesized that the timing of S-phase entry is regulated by 2 CCGs, *p20* and *p21*, which control tissue specific S-phase timing in zebrafish.^8^ To identify which of these inhibitors is the most abundant in skin we compared their expression across a LD cycle in skin samples and found that *p20* is significantly more abundant than *p21* in this particular tissue (**Fig. 4A**). The circadian profile of *p20* expression showed robust rhythms in skin samples, peaking in the night at ZT15 (**Fig. 4B**). We have previously reported similar findings in zebrafish embryos, where *p20* is the main regulator in the developing brain. In the developing zebrafish larvae *p20* shows a clear phase difference in expression compared to *p21*, where it is responsible for timing actual S-phase events to ZT3, in the early day.^8^ This peak at ZT3 in S-phase would be highly appropriate in skin, as it would coincide with the peak in expression of DNA repair genes, therefore possibly providing protection against UV light-induced mutations during the process of DNA replication. Surprisingly, tumor samples revealed a similar *p20* expression pattern in LD, suggesting that the regulation of S-phase timing is not disrupted in tumors unlike the regulation of mitosis. However, rhythms in *p20* in DD do become less precise over time in tumors, which may be a reflection of the somewhat disrupted circadian pacemaker in these cells. Other S-phase genes such as *p21*, *cdk2* and *PCNA* showed an up-regulation in expression in tumor samples in LD and DD, with a loss of rhythmicity in DD for *cdk2* and *PCNA* compared to skin samples (**Fig. 4B**, **Tables S5, S6**). We can conclude that the cell cycle in melanoma tumors displays disrupted circadian properties with a loss in rhythmicity of several genes, especially those involved in mitosis.

We have demonstrated in this study that light-inducible genes, such as *cry1a* and *per2*, showed a reduced response to light in zebrafish melanoma compared to healthy skin. This impaired light response also resulted in a total loss in expression of light-induced DNA damage repair genes during the daytime, when UV-induced mutation risk is at its highest. We believe that this impaired light response, observed in tumors, could reduce the strength of entrainment of the clock and may lead to the reduced amplitude in clock gene oscillations in LD. The lower level of expression of clock genes in tumors is also likely to reduce the level of precise control over cellular clock outputs, such as the cell cycle. Key cell cycle genes show a loss of circadian regulation, and mitotic events consequently are also no longer rhythmic, meaning that cell division occurs to a greater extent during the day compared to healthy skin. The coupling process between the circadian clock and control of mitosis is clearly disrupted in melanoma. Loosing the ability to repair DNA combined with incorrect circadian timing of cell cycle events is likely to create a highly mutagenic environment for cells in the tumor and promote tumourigenesis even further. Genomic instability and sustained proliferative signaling are cancer hallmarks.^50^ We therefore identified a crucial role for the zebrafish skin clock, which is to time the cell cycle to periods of darkness or times of highest active DNA repair in order to confer protection from UV in dividing cells.

What might be the cause of this decoupling between the cellular clock and the timing of mitotic events within the melanoma? Light is typically believed to be the dominant entraining signal for the circadian clock. However, glucocorticoid signaling has also been shown to play a key role in entrainment, especially of peripheral circadian pacemakers.^51^ Moreover, the presence of glucocorticoid signaling/cortisol has been shown to be important for the control of rhythmic cell cycle events in healthy tissues during zebrafish embryo development.^52^ In the case of the strong *rx3* zebrafish mutants, which possess fewer corticotropes within the anterior pituitary and reduced levels of cortisol, the molecular circadian clock appears to be normal, but there is a strong reduction in the amplitude of the circadian rhythm in S-phase, as measured by BrdU incorporation into the skin. Tonic treatment of strong *rx3* mutant larvae with the glucocorticoid receptor agonist, dexamethasone, can largely rescue this cell cycle rhythm, re-establishing the coupling between clock and cell cycle. These results have some clear similarities with the data we have obtained in melanoma. Though the clock in the tumor is somewhat more disrupted, especially in terms of the light input pathway, it is still able to show clear daily molecular oscillations, yet it is the coupling to control mitotic rhythms that is clearly lost. This raises the interesting possibility that there maybe a disruption in glucocorticoid signaling in the context of the melanoma tumor. This, of course, could occur at many levels from disruption of glucocorticoid receptors in the tumor, through to perturbation of cortisol levels in an unhealthy animal. Clearly, future studies will need to explore the possible role played by glucocorticoid signaling in coupling clock and cell cycle, especially during the process of tumourigenesis. Zebrafish melanoma certainly offers a unique model in which to study the intricate link between the circadian clock, signaling pathways and the cell cycle.

Revealing how clock-cell cycle interactions function in a cancer environment is an important issue, not only to understand the circadian biology of cancer, but also to provide insights on how to improve cancer treatment through chronotherapy. Chronotherapeutic regimes, delivering drugs at specific times of the day for optimal drug action and reduced toxic side effects, have already shown promising results in metastatic prostate cancer patients in a timed regime compared to conventional treatment.^21^ The circadian profile of cell cycle genes generated in this study clearly points to an asynchrony in melanoma tumors compared to healthy skin cells. In such a scenario, the potential exists, therefore, to apply drugs to kill cancer cells at a time where there will be minimal impact on healthy tissue. Zebrafish have already been described as an excellent model for pharmacological studies and future experiments will assess the efficacy of key melanoma drugs within a chronochemotherapeutic regime.

## Materials and Methods

### Animal husbandry

*Tg(mitfa:V12Ras)*; *mitfa*^−/−^ animals were obtained from Dr. Adam Hurlstone (University of Manchester) and were raised and maintained in the zebrafish animal facility of University College London, as previously described.^53^ All animals were held in a Home Office approved animal facility and in accordance with Home Office regulations regarding animal maintenance and care. Animal handling has been approved by a UCL ethics committee and meets all of the requirements of the Animal Welfare Act of 2006. Individual experiments were performed under animal license number PIL 40/3292. Animals were sacrificed in accordance with Schedule 1 of the Animal Welfare Act of 2006, to ensure minimal suffering. Adult fish were kept in light cabinets and exposed to a lighting regime of 14L/10D unless stated otherwise.

### Generation of transgenic melanoma fish

The miniCoopR GFP vector was a gift from Adam Hurlstone (University of Manchester).^36^ 75pg of miniCoopR-GFP vector and 75pg of *tol2* transposase mRNA were microinjected into one-cell stage embryos generated from a *(mitfa:Ras^V12^); ^mitfa−/−^* × ^mitfa−/−^ zebrafish cross. Transgenic animals recapitulated a melanoma tumor phenotype observed in the *Tg(mitfa:Ras*^V12^*)*,^35^ but with an earlier onset of 4 weeks of age and with far greater penetrance as all fish developed tumors. Animals developing either melanoma tumors or normal pigmentation were used for tumor and skin sample collection, respectively. It should be noted that the tumor bearing fish are culled before the size of the tumor affect their feeding and swimming, so the presence of the tumor does not affect fish survival.

### Quantitative PCR (qPCR)

Adult skin and tumor samples were harvested at the indicated zeitgeber or circadian time (ZT or CT, where ZT0 equals lights on). RNA extraction, cDNA synthesis and qPCR experiments were carried out as previously described.^8^ ΔCT was calculated using *ribosomal 18S* as a reference gene. Relative expression levels were plotted after determining ΔΔCt by normalizing to a single sample with a high ΔCT value. Primer sequences are listed in **Table S7**.

### Bioluminescence assays

Skin and tumor samples from *V12Ras*^+^; *GFP*^+^ and *GFP*^+^ injected animals in a *per3-luciferase* background were dissected and placed in medium containing 0.5mM of luciferin (Promega) in a 96-well plate. Samples were maintained at 28°C on a light-dark (LD) cycle (12L:12D) and transferred into either constant darkness (DD) or a reverse light/dark cycle. Bioluminescence was monitored on a Packard TopCount NXTscintillation counter. The luminescent rhythm parameters (phase and amplitude) were calculated after detrending by subtracting a 24-h moving average from the raw data.

### Phospho-Histone H3 immunohistochemistry

Whole zebrafish were fixed at specified time points on a LD cycle^33^ and sectioned as previously described^49^ with the following changes. Animals were fixed in 4% PFA in 0.1 phosphate buffer at 4°C for 5 days, then transferred to a 0.25 M EDTA solution for 3 days at room temperature. EDTA was rinsed off with water before immersing the samples in 30% sucrose solution for an additional 2 days. Tumor-bearing and healthy fish were cryosectioned at 10μm and stained, as previously described.^49^ Images were collected using a Zeiss AxioScan Z1 slide scanner. Quantification of pH3-positive cells was calculated relative to the total number of cell nuclei in skin layers, stained with DAPI and within the GFP-labeled tumor area.

### Statistical analysis

The data in this study are presented as the mean ± SEM (n ≥ 3). Statistical significance was determined by an unpaired 2-tailed Student t-test or analysis of variance (ANOVA), followed by Newman–Keuls multiple comparison post-test in GraphPad Prism.

### Light pulse experiments

Fish were kept on a LD cycle for 7 days before being transferred to DD. At CT16 on the first DD night, animals were exposed to a 3 h light pulse using LED lights with an average intensity of 65 μW/cm^2^.

### Rhythm analysis

Rhythm analyses were performed by the Cosinor method (Nelson et al. 1979), using the El Temps software developed by Prof. A. Díez Noguera. Rhythms are considered significant when *P* < 0.05. *P < 0.05; **P < 0.01; ***P < 0.001 and non-significant rhythms when *P* < 0.1.

## Supplemental Material

Supplemntary Materials
