# Supplementary material for: Impaired light detection of the circadian clock in a zebrafish melanoma model
Source: Cell Cycle. 2015 Apr 2;14(8):1232–41. doi: 10.1080/15384101.2015.1014146 (PMC4615116; doi:10.1080/15384101.2015.1014146)
Supplement: Supplemntary Materials [file kccy-14-08-1014146-s001.pdf]

SUPPLEMENTARY FIGURES

Figure S1

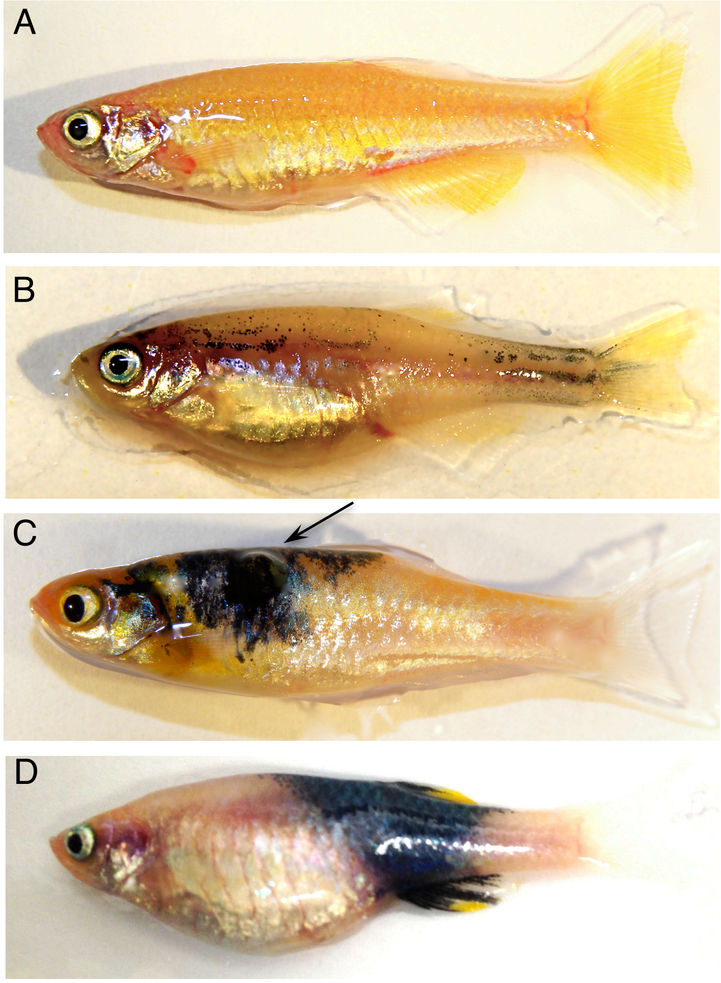

Tg(mitfa:V12Ras) x *mitfa*$^{-/-}$

mitfa$^{-/-}$
+ miniCoopR-GFP

Tg(mitfa:V12Ras) x *mitfa*$^{-/-}$
+ miniCoopR-GFP with
tumour

Tg(mitfa:V12Ras) x *mitfa*$^{-/-}$
+ miniCoopR-GFP with
neavus

Figure S1
(A) Adult V12Ras transgenic melanoma zebrafish in the *mitfa*$^{-/-}$ background showing no melanocyte pigmentation. (B) Adult non V12Ras transgenic zebrafish after injection with the miniCoopR-GFP plasmid showing rescued normal melanocyte pigmentation. (C-D) Adult V12Ras transgenic melanoma zebrafish after injection of miniCoopR-GFP plasmid showing rescued in pigmentation of melanocytes over expressing V12Ras. Tumour marked with a black arrow.

Figure S2

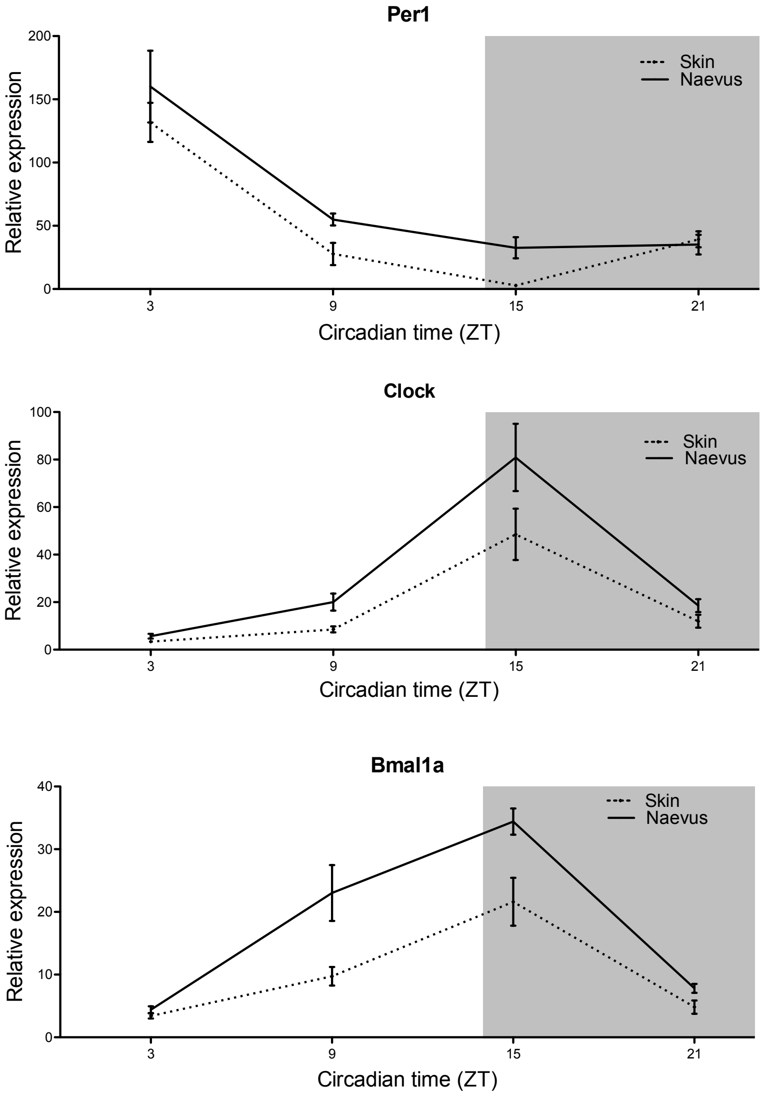

Figure S2
qPCR analysis of core clock genes *per1*, *clock1*,*Bmal1a* in skin and naevus in LD. Data represent the mean ± SEM of minimum 5 samples per time point. White and gray backgrounds represent light and dark phases respectively.

Figure S3

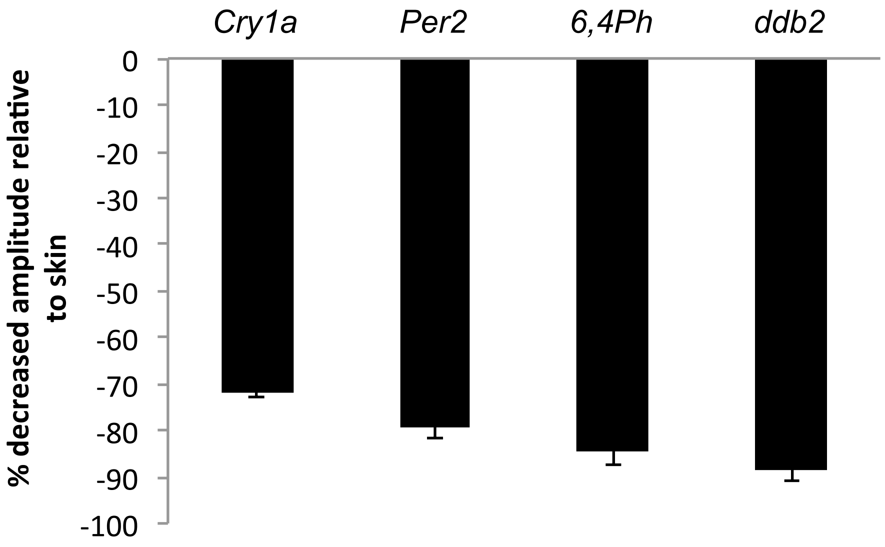

Figure S3
Quantification of the reduction in amplitude of expression of *cry1a*, *per2*, *6,4ph* and *ddb2* in tumour using skin as a reference. Data represent the mean ± SEM of 5 samples.

Figure S4

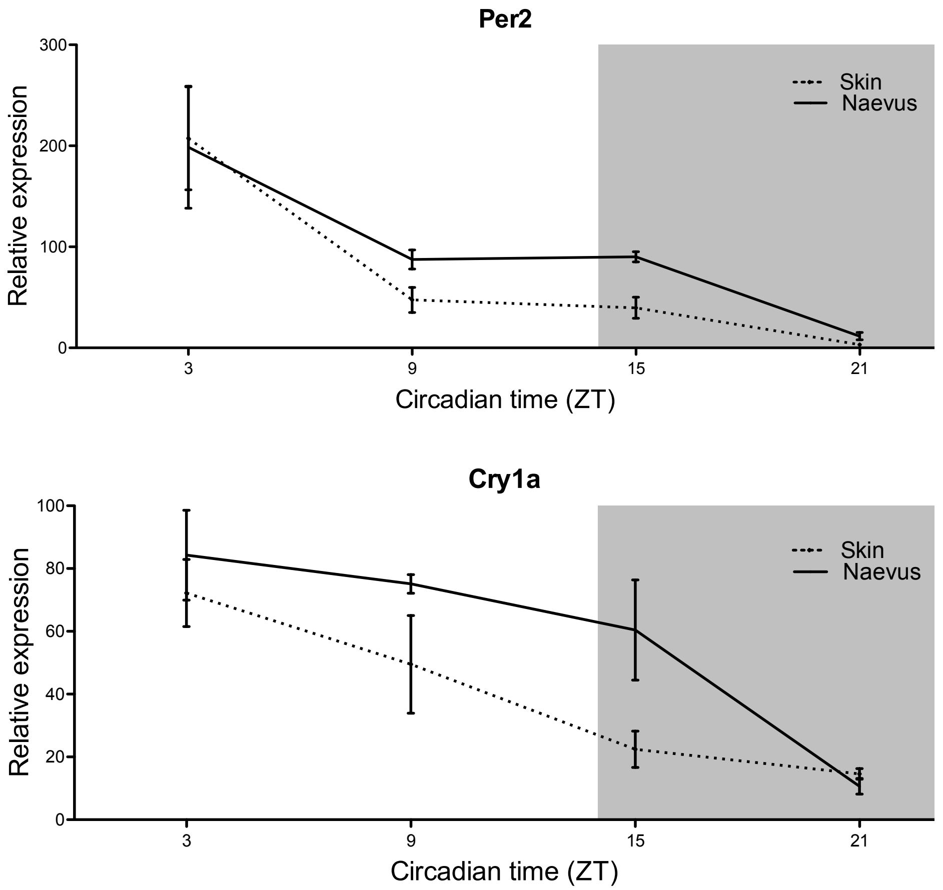

Figure S4
qPCR analysis of core clock genes *per2* and *cry1a* in skin and naevus in LD. Data represent the mean ± SEM of minimum 5 samples per time point. White and gray backgrounds represent light and dark phases respectively.

Figure S5

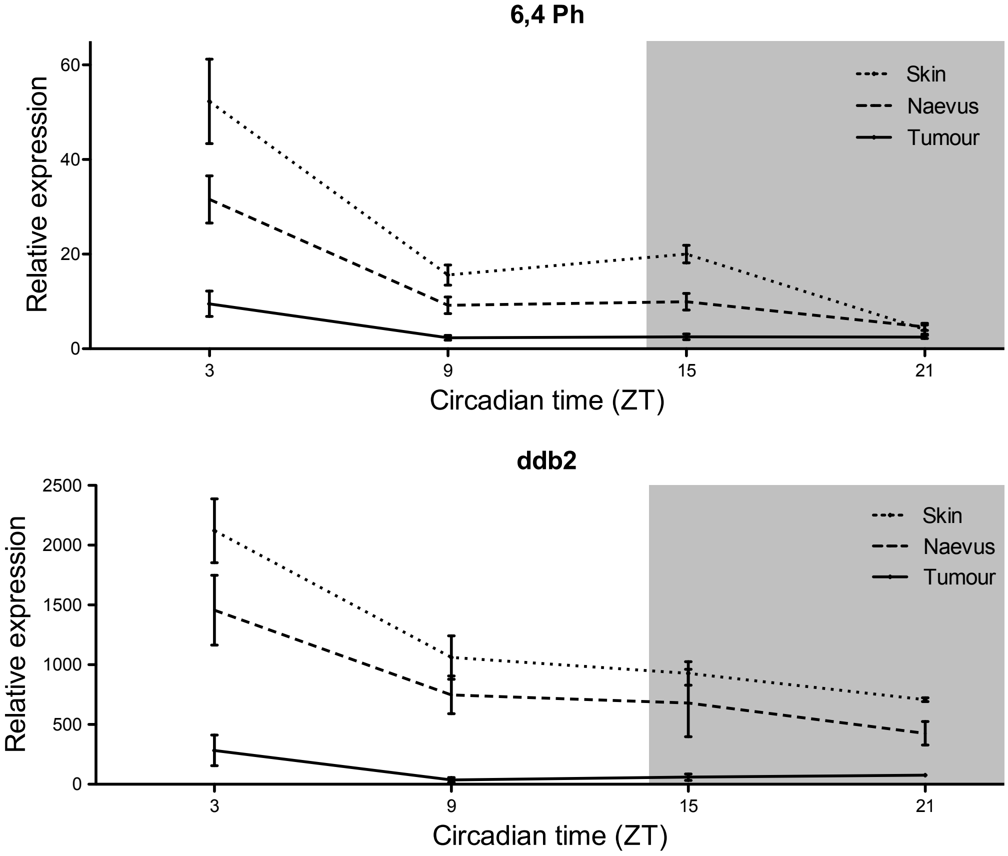

Figure S5
qPCR analysis of core clock genes *6,4ph* and *ddb2* in skin and naevus in LD. Data represent the mean ± SEM of minimum 5 samples per time point. White and gray backgrounds represent light and dark phases respectively.

Figure S6

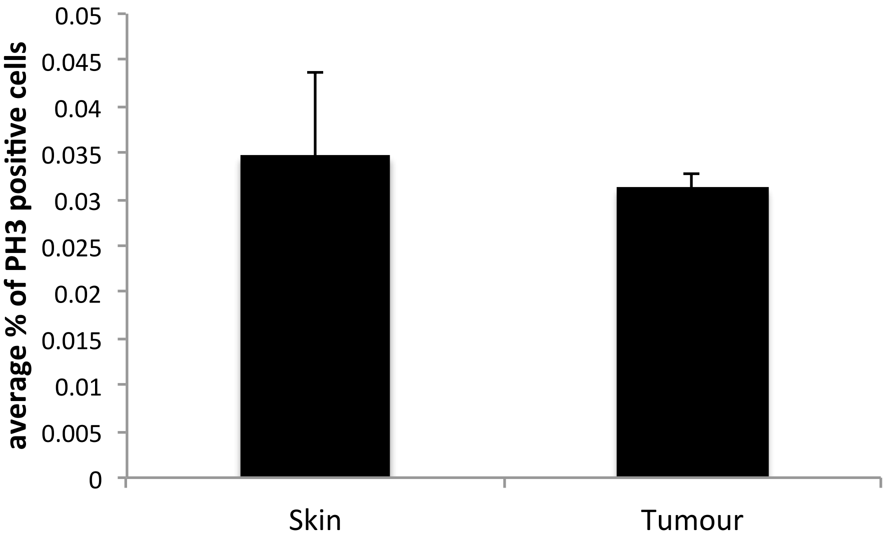

Figure S6
Average of % of PH3 positive cells undergoing mitosis across ZT3, ZT9, ZT15 and ZT21 in both skin and tumour.

Table S1

Cosinor analysis for circadian clock gene expression profiles in LD and DD.

Gene			Mesor (95% CI)	Amplitude (95% CI)	Acrophase (95% CI)	A/M	Rhythmic (p)
Per1	LD	Skin	46.82 (39.2~54.3)	56.96 (43.3~70.5)	2.83 (1.9~3.7)	1.22	Yes ***
		Tumour	36.20 (30.7~41.6)	27.65 (17.9~37.3)	4.73 (3.4~6.0)	0.76	Yes ***
	DD	Skin	33.17 (28.3~38.0)	24.47 (15.6~33.3)	4.66 (3.3~6.0)	0.74	Yes ***
		Tumour	41.86 (35.4~48.2)	26.07 (14.8~37.2)	6.23 (4.5~8.0)	0.62	Yes ***
Bmal1a	LD	Skin	45.05 (37.2~52.8)	43.08 (29.7~56.4)	13.79 (12.5~15.1)	0.96	Yes ***
		Tumour	41.48 (34.5~48.4)	20.80 (8.1~33.4)	13.88 (11.4~16.2)	0.87	Yes ***
	DD	Skin	19.37 (15.9~22.7)	16.80 (10.7~22.8)	17.95 (16.5~19.3)	0.87	Yes ***
		Tumour	32.30 (26.7~37.8)	19.92 (10.1~29.6)	16.63 (14.6~18.6)	0.50	Yes**
Clock	LD	Skin	35.04 (25.3~44.7)	45.49 (28.9~62.0)	15.34 (13.8~16.8)	1.30	Yes ***
		Tumour	19.05 (15.2~22.8)	10.26 (3.1~17.4)	14.66 (11.9~17.3)	0.54	Yes *
	DD	Skin	12.91 (10.0~15.8)	12.45 (7.2~17.6)	18.49 (16.9~20.1)	0.96	Yes ***
		Tumour	15.89 (13.2~18.5)	10.75 (5.9~15.5)	17.47 (15.7~19.2)	0.68	Yes ***
Per2	LD	Skin	78.32 (51.8~104)	71.7 (28.3~115)	3.01 (0.2~5.7)	0.92	Yes **
		Tumour	28.42 (24.4~32.4)	8.83 (1.9~15.8)	6.48 (3.2~10.3)	0.31	Yes *
	DD	Skin	5.23 (4.2~6.2)	-	-	-	ns (0.66)
		Tumour	23.04 (16.8~29.2)	12.58 (1.6~23.4)	15.96 (11.8~19.9)	0.55	Yes *
Cry1a	LD	Skin	36.42 (29.3~43.4)	31.79 (19.7~43.8)	5.04 (3.3~6.5)	0.87	Yes ***
		Tumour	13.13 (11~15.2)	9.13 (5.6~12.6)	6.20 (4.4~7.8)	0.70	Yes ***
	DD	Skin	10.18 (7.9~12.4)	8.17 (3.9~12.3)	4.11 (2.2~6.1)	0.80	Yes ***
		Tumour	8.72 (6.7~10.6)	5.96 (2.5~9.3)	5.49 (3.0~7.8)	0.68	Yes **

Table S1. Data represent the mesor and amplitude in relative expression to reference gene, the acrophase in circadian time and their respective 95% confidence intervals, the amplitude relative to the mesor (A/M) and the significance of rhythmicity (*p<0.05; **p<0.01; ***p<0.001) or non-significance with the p value indicated between brackets for skin and tumours in LD and DD for clock genes.

Table S2

Comparisons between the parameters of the Cosinor analysis for clock genes between samples skin and tumour and conditions LD and DD.

	% change in tumour relative to skin		% change in DD relative to LD	
	**LD**	**DD**	**Skin**	**Tumour**
Per1				
Mesor	-22.68	26.19	-29.14	15.65
Amplitude	-51.45	6.53	-57.04	-5.73
A/M	-37.21	-15.58	-39.37	-18.49
Bmal1a				
Mesor	-57.00	114.14	-57.00	114.14
Amplitude	-60.99	23.75	-60.99	23.75
A/M	-9.27	-42.21	-9.27	-42.21
Clock				
Mesor	-45.63	23.02	-63.13	-16.58
Amplitude	-77.44	-13.63	-72.63	4.80
A/M	-58.51	-29.79	-25.77	25.64
Per2				
Mesor	-63.71	339.90	-93.31	-18.92
Amplitude	-87.68	-	-	42.53
A/M	-66.05	-	-	75.78
Cry1a				
Mesor	-63.93	-14.38	-72.03	-33.62
Amplitude	-71.28	-27.09	-74.27	-34.69
A/M	-20.39	-14.85	-8.01	-1.61

Table S3

Cosinor analysis for DNA-damage repair gene expression profiles in LD and DD

Gene			Mesor (95% CI)	Amplitude (95% CI)	Acrophase (95% CI)	A/M	Rhythmic (p)
6,4 PH	LD	Skin	23.56 (17.8~29.2)	21.26 (11.6~30.9)	4.29 (2.2~6.1)	0.90	Yes ***
		Tumour	4.71 (3.5~5.8)	2.27 (0.2~4.3)	-	0.48	ns (0.28)
	DD	Skin	7.84 (6.2~9.4)	-	-	-	ns (0.75)
		Tumour	4.88 (3.5~6.1)	2.06	-	0.42	ns (0.12)
ddb2	LD	Skin	1071 (876~1265)	686 (346~1025)	2.63 (0.6~4.6)	0.64	Yes ***
		Tumour	112.49 (73~151)	60.84	-	0.54	ns (0.96)
	DD	Skin	381.3 (289~473)	99.7	-	0.26	ns (0.24)
		Tumour	49.64 (31.2~68.0)	16.44	-	3.36	ns (0.49)

Table S3. Data represent the mesor and amplitude in relative expression to reference gene, the acrophase in circadian time and their respective 95% confidence intervals, the amplitude relative to the mesor (A/M) and the significance of rhythmicity (*p<0.05; **p<0.01; ***p<0.001) or non-significance with the p value indicated between brackets for skin and tumours in LD and DD for DNA damage repair genes.

Table S4

Comparisons between the parameters of the Cosinor analysis for DNA damage repair genes between samples skin and tumour and conditions LD and DD.

	**% change in tumour relative to skin**		**% change in DD relative to LD**	
	**LD**	**DD**	**Skin**	**Tumour**
6,4 PH				
Mesor	-79.98	-37.68	-66.71	3.60
Amplitude	-89.30	-	-	-9.24
A/M	-46.54	-	-	-12.40
ddb2				
Mesor	-89.50	-98.72	-64.40	-95.66
Amplitude	-91.13	-83.51	-85.47	-72.97
A/M	-15.53	1187.04	-59.17	522.08

Table S5

Cosinor analysis of circadian rhythmicity of qPCR cell cycle gene expression in LD and DD.

Gene			Mesor (95% CI)	Amplitude (95% CI)	Acrophase (95% CI)	A/M	Rhythmic (p)
cyclinB1	LD	Skin	33.54 (24.8~42.2)	27.10 (12.9~41.4)	16.91 (14.3~19.1)	0.81	Yes ***
		Tumour	23.10 (18.5~27.6)	8.91 (0.2~17.6)	15.70 (10.8~21)	0.39	Yes *
	DD	Skin	10.54 (9.1~11.9)	4.86 (2.3~7.4)	17.54 (15.4~19.6)	0.46	Yes**
		Tumour	20.33 (15.3~25.2)	5.01	-	0.25	ns (0.27)
cdk1	LD	Skin	12.04 (7.6~16.4)	13.47 (6.3~20.6)	15.37 (12.8~17.7)	1.12	Yes ***
		Tumour	12.46 (9.6~15.2)	6.93 (1.5~12.3)	16.01 (13.1~19.3)	0.56	Yes**
	DD	Skin	4.72 (3.8~5.5)	2.74 (1.1~4.3)	16.88 (14.6~19.2)	0.58	Yes *
		Tumour	14.71 (10.3~19.1)	3.03	-	0.21	ns(0.60)
wee1	LD	Skin	4.86 (3.7~5.9)	2.79 (1.0~4.5)	15.50 (12.5~18.4)	0.57	Yes **
		Tumour	6.01 (4.9~7.1)	0.87	-	0.14	ns
	DD	Skin	2.51 (2.1~2.9)	0.21	-	0.08	ns
		Tumour	6.58 (5.2~7.9)	1.26	-	0.19	ns (0.48)
p20	LD	Skin	41.02 (29.7~52.2)	45.54 (27~64)	15.71 (13.8~17.4)	0.90	Yes ***
		Tumour	80.61 (63.4~97.8)	78.7 (45~112)	15.54 (14.1~17)	0.98	Yes ***
	DD	Skin	48.37 (37.8~58.9)	56.23 (37.1~75.2)	15.81 (14.5~17.1)	1.16	Yes ***
		Tumour	88.03 (71.6~104)	47.86 (19.2~76.5)	17.20 (14.7~19.5)	0.54	Yes **
p21	LD	Skin	27.37 (22.3~32.3)	25.98 (17.0~34.9)	19.11 (17.7~20.4)	1.05	Yes ***
		Tumour	39.78 (31.3~48.2)	30.12 (15.5~44.6)	20.69 (18.6~22.6)	0.76	Yes ***
	DD	Skin	23.77 (16.8~30.6)	27.73 (16.2~39.2)	20.81 (18.9~22.6)	1.17	Yes ***
		Tumour	56.25 (43.3~69.1)	16.73	-	0.30	ns (0.16)
cdk2	LD	Skin	9.50 (7.1~11.9)	5.73 (1.6~9.8)	13.85 (10.7~17.2)	1.66	Yes *
		Tumour	19.20 (15.8~22.5)	1.08	-	0.06	ns
	DD	Skin	5.32 (4.4~6.2)	1.48	-	0.28	ns
		Tumour	19.90 (16.5~23.1)	4.14	-	0.21	ns (0.17)
PCNA	LD	Skin	8.20 (6.6~9.7)	3.26 (0.4~6.1)	7.07 (2.8~10.5)	0.40	Yes *
		Tumour	14.83 (11.6~18.0)	3.43	-	0.23	ns
	DD	Skin	6.88 (5.7~8.0)	1.56	-	0.23	ns
		Tumour	18.78 (13.9~23.6)	3.46	-	0.18	ns (0.61)

Table S5. Data represent the mesor and amplitude in relative expression to reference gene, the acrophase in circadian time and their respective 95% confidence intervals, the amplitude relative to the mesor (A/M) and the significance of rhythmicity (*p<0.05; **p<0.01; ***p<0.001) or non-significance with the p value indicated between brackets for skin and tumours in LD and DD for cell cycle genes

Table S6
Comparisons between the parameters of the Cosinor analysis for cell cycle genes between samples skin and tumour and conditions LD and DD.

		**% change in tumour relative to skin**		**% change in DD relative to LD**	
		**LD**	**DD**	**Skin**	**Tumour**
CyclinB1					
	Mesor	-31.16	92.84	-68.56	-11.93
	Amplitude	-67.11	2.97	-82.04	-43.78
	A/M	-52.23	-46.60	-42.88	-36.16
cdk1					
	Mesor	3.53	211.46	-60.76	18.03
	Amplitude	-48.51	10.45	-79.64	-56.33
	A/M	-50.26	-64.54	-48.11	-63.00
wee1					
	Mesor	23.78	162.08	-48.29	9.48
	Amplitude	-68.84	-	-92.48	44.83
	A/M	-74.83	128.94	-85.45	32.28
p20					
	Mesor	96.50	81.98	17.93	9.22
	Amplitude	72.76	-14.88	23.47	-39.17
	A/M	8.36	-53.23	29.03	-44.30
p21					
	Mesor	45.36	136.62	-13.14	41.40
	Amplitude	15.92	-39.68	6.73	-44.47
	A/M	-28.11	-74.51	10.77	-60.73
cdk2					
	Mesor	102.11	273.17	-43.91	3.55
	Amplitude	-81.16	179.73	-74.19	283.33
	A/M	-96.60	-25.04	-83.23	270.18
PCNA					
	Mesor	81.36	172.79	-15.80	26.66
	Amplitude	4.91	121.79	-52.29	0.87
	A/M	-42.15	-18.69	-43.33	-20.36

Table S7 qPCR primer sequences

Genes		qPCR primer sequence
18s	3'	TCGCTAGTTGGCATCGTTTATG
	5'	CGGAGGTTCGAAGACGATCA
per1	3'	ATCCAGACCCCAATACAAC
	5'	GGGAGACTCTGCTCCTTCT
clock1	3'	GCTGCAGTTTTCCACACAGA
	5'	ACCCTGTCCTTGAACCCTCT
bmal1a	3'	AGGGAAACGGCCTCTTCA
	5'	GCGTGGCAGTGATGTTTAA
cry1a	3'	AGGCTTACACAGCAGCATCA
	5'	CTGCACTGCCTCTGGACTTT
per2	3'	TGGCTCTGGACAGAAGTGAG
	5'	GGATGTCTCGAGAAGGCAAC
p21	3'	CCGCATGAAGTGGAGAAAAC
	5'	ACGCTTCTTGGCTTGGTAGA
p20	3'	GGTCCGTGTGGACTTGATTT
	5'	CCTCTTCAACAGCCCATGAT
pcna	3'	GGCAAGATCAAGCTCTCACA
	5'	TGCACTGGCTCATTCATCTC
cyb1	3'	GTACCCACCAGAGATTGCAG
	5'	GGTAGAGGCCTTCCAAAACC
wee1	3'	GACCAAAATGCACTTCGTGA
	5'	TGCCCAGGCAGAATAATACC
cdk2	3'	GGCTGCATCTTTGCTGAAAT
	5'	GAGTGCCCAGTGTTCGAAAT
6.4ph	3'	TGTGGATCATGAGGTTGTCC
	5'	TTGATGGATGGACTCGCTTT
ddb2	3'	GAGGCAGAGCTGGAGGTTC
	5'	TCGGTCTTGCTCTTGGTCTT
